# Hub Occupancy by Competitively Interacting Proteins Obeys a Simple Queuing Law

**DOI:** 10.1021/acs.jpcb.5c04305

**Published:** 2025-09-24

**Authors:** Yuming Jiang, Antun Skanata, Liviu Movileanu

**Affiliations:** † Department of Physics, 2029 Syracuse University, 201 Physics Building, Syracuse, New York 13244-1130, United States; ‡ The BioInspired Institute, Syracuse University, Syracuse, New York 13244, United States; § Department of Biomedical and Chemical Engineering, Syracuse University, 329 Link Hall, Syracuse, New York 13244, United States; ∥ Department of Biology, Syracuse University, 114 Life Sciences Complex, Syracuse, New York 13244, United States

## Abstract

Coordinated interactions between a protein hub, or receptor, and its cognate protein ligands are at the heart of cell signaling. Any significant perturbations in their kinetic and dynamic complexities result in major alterations in biochemical traffic at the subcellular and extracellular levels. The coexistence of multiple ligands with varying local concentrations and affinity constants, as well as the transient nature of their underlying protein–protein interactions (PPIs), makes predicting hub occupancy a challenging task. Here, we develop models of PPIs anchored in queuing theory to determine hub occupancy as a function of the kinetic rate constants and concentrations in complex mixtures of protein ligands. We find that in a ternary mixture of protein ligands spanning a range of kinetic rate constants, the concentration of one ligand can significantly influence the competitive PPIs between the other two ligands and the protein receptor, thereby impacting its overall occupancy. Further, for more complex mixtures, we developed a coarse-graining approach to compartmentalize large numbers of ligands competing for the same binding site of the receptor. Our analytical strategy provides a mechanistic and quantitative understanding of competitive PPIs, with broad applicability to biochemical processes, protein analytics, and drug development.

## Graphical abstract

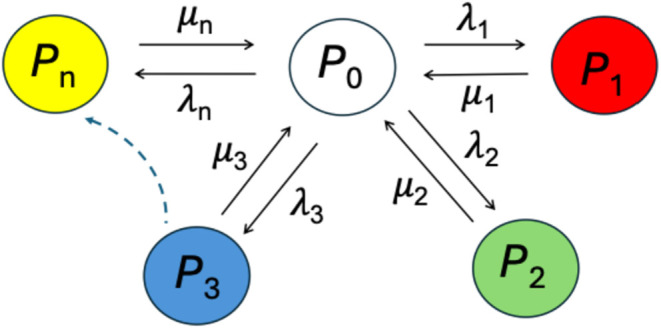

## Introduction

Complex networks of interactions among numerous proteins determine a wide range of cellular functions. Specific physical and biochemical stimuli arise from transient binary associations of proteins, known as reversible protein–protein interactions (PPIs).^1,2^ The discovery of the human proteome has sparked extensive structural, computational, and functional studies of PPIs, many of which are mediated by multitasking binding sites.^3–7^ Currently, we are aware of large networks of hub-controlled protein–protein interactions (PPIs), referred to as interactomes.^5,8–13^ For example, c-myelocytomatosis (MYC), a transcription factor with implications in cancer progression and development, reversibly interacts with over 300 binding proteins, employing one of its several evolutionarily conserved homologous boxes.^9,14,15^ In another example, WD40-repeat protein 5 (WDR5), which is involved in modulating gene expression and cell development, features two binding sites that facilitate binary physical interactions with dozens of proteins.^5,16^ The complex interactomes of intracellular proteins are extended to extracellular proteins. For instance, multiple growth factor ligand proteins interact with the epidermal growth factor receptor (EGFR), regulating its signaling activity.^17,18^

Several isoforms of individual interacting proteins exacerbate the complexity of the structure, composition, and functional roles of interactomes. As an example, six mixed lineage leukemia (MLL/SET1) polypeptides interact with WDR5 through one of its binding sites.^4,19,20^ In addition, post-translational modifications of proteins in the binding sites, which result in significantly altered affinities, amplify the complications of the entanglement of multiple protein ligands interacting with the same receptor. On the one hand, traditional approaches in the bulk aqueous phase are not suitable for detecting and characterizing weakly interacting proteins, which result from either short binding durations or rare binding events, or both.^6,21,22^ On the other hand, single-molecule approaches exhibit an extensive time bandwidth and resolution that can enable evaluations of these unusually weak interactions.^23–30^ However, many single-molecule technologies have yet to achieve widespread adoption. Hence, numerous regions and individual maps of human interactomes remain uncharted.^11^ These complexities are also enhanced by individual subpopulations of binding interactions between two proteins, which are generated by potential multimodal PPIs.^15,29,31^ Yet, a heterogeneous distribution of binding times in the form of different event subpopulations is likely detectable by single-molecule technologies, as they do not involve averaging over an ensemble of molecules.^32^ Despite significant progress in understanding large-scale interactomes and reversible PPIs, there is a pressing need to develop robust experimental and computational methods for assessing the impact of competitive PPIs on the activity of a protein hub or receptor.

Recently, we developed a single-molecule nanopore-based approach to examine competitive PPIs in binary mixtures of protein ligands against a protein receptor.^33^ In these mixtures, we measured receptor occupancy as a function of the concentrations of the two ligands involved. For certain combinations of kinetic rate constants, we observed a nonmonotonic dependence of the experimental receptor occupancy on the ligand concentration. Existing kinetic models do not account for this surprising result.^34,35^ We then discovered that a simple model anchored in a mathematical theory of queuing processes^36,37^ can be readily utilized to account for the biphasic dependence of receptor occupancy on varying ligand concentrations. Moreover, we found that this queuing model accurately predicts the experimentally observed ligand dependencies with no adjustable fit parameters. Currently available models of enzyme kinetics operate over a concentration range that is not supported by single-molecule experiments, which are otherwise opportunistic alternatives for evaluating receptor occupancy at single-molecule precision.

Motivated by these recent developments, we extend our analytical approach to situations involving multiple protein ligands interacting with a single protein receptor. We illustrate the applicability of our method by presenting three distinct examples that highlight the nontrivial aspects of receptor occupancy resulting from competitive PPIs. In the first example, we test a system with three protein ligands of varying binding affinities simultaneously interacting with a protein receptor. We find that the concentration of one of the ligands can significantly modulate the partial receptor occupancies of the other two ligands, thereby altering their competitive interactions with the receptor. In the second example, we employ a coarse-graining analytical approach to analyze the system, which involves five proteoforms competitively interacting with a protein hub that is part of a larger epigenetic complex. In the third example, we employ a binary mixture of two ligands, one of which undergoes a post-translational modification, resulting in a high binding affinity with the protein receptor. Our queuing model, a stochastic framework of probabilistic waiting lines, provides key information on the implications of significant alterations in the kinetics of one PPI on the partial receptor occupancies by individual interacting participants. Taken together, these kinetic evaluations contribute to a better quantitative understanding of the complex changes in the activity of a specific hub resulting from biochemical modifications of one of its interacting partners.

## Methods

To describe the molecular process of binding and unbinding events to a protein receptor, we consider a model in which the arrival of a protein ligand to the protein receptor follows a Poisson process with a rate λ. The rate of service, μ, models the rate of release of a captured ligand. Thus, the probability that the protein receptor is found in a bound state at any moment in time is (see Supporting Methods)­

1 $$P=\frac{\lambda }{\lambda +\mu }$$

In solutions containing a single ligand type at a concentration *c* and with the rate constants of association and dissociation, *k*_on_ and *k*_off_, respectively, eq 1 with λ = [*c*] *k*_on_ and μ = *k*_off_ exactly reproduces receptor occupancy obtained from kinetic models and predicts experimental data to high accuracy.^33^ In a binary mixture of protein ligands, a protein receptor can exist in one of three possible states: unbound, bound to ligand 1 (L_1_), or bound to ligand 2 (L_2_), with probabilities *P*_0_, *P*_1_, or *P*_2_, respectively. In a quasi-steady state, fluxes to each of the occupied states are balanced by the total flux to the unoccupied state, as follows

2 $$\mu_{1}P_{1}+\mu_{2}P_{2}=\left(\lambda_{1}+\lambda_{2}\right)P_{0}$$

where the forward and reverse rates are denoted by λ*_i_* and μ*_i_* for ligand *i*, respectively. Here, λ*_i_* = [*c_i_*]*k*_on,*i*_ and μ*_i_* = *k*_off,*i*_. For each protein ligand, we determine *P*_*i*_ through eq 1 and solve eq 2 for *P*_0_. (see Supporting Methods for derivation). Receptor occupancy is then

3 $$O=1-P_{0}=1-\frac{\frac{\mu_{1}\lambda_{1}}{\mu_{1}+\lambda_{1}}+\frac{\mu_{2}\lambda_{2}}{\mu_{2}+\lambda_{2}}}{\lambda_{1}+\lambda_{2}}$$

Here, we highlight that noninteracting quantities are denoted as *P*, and quantities that contain information about competitive PPIs are denoted as O . The probabilities *P_i_* < 1 we use to solve eq 2 are obtained for single-ligand PPIs, and therefore do not contain the competitive interactions. In this model, the competitive interactions arise through the flux balance constraint, which is provided in eq 2. This ensures that the total occupancy remains less than 1 (Supporting Methods).

Generally, when there exist *n* ligand types, receptor occupancy is given by (Supporting Methods)­

4 $$O=1-\frac{\frac{\mu_{1}\lambda_{1}}{\mu_{1}+\lambda_{1}}+\frac{\mu_{2}\lambda_{2}}{\mu_{2}+\lambda_{2}}+...+\frac{\mu_{n}\lambda_{n}}{\mu_{n}+\lambda_{n}}}{\lambda_{1}+\lambda_{2}+...+\lambda_{n}}$$

By requiring proportional fluxes along each branch, we rewrite this in terms of partial occupancies

5 $$O=O_{1}+O_{2}+...+O_{n}$$

where

6 $$O_{i}=\frac{\lambda_{i}\prod_{j\neq i}\mu_{j}}{\sum_{i=1}^{n}\lambda_{i}\prod_{j\neq i}\mu_{j}}O,i=1,2,...,n$$

is the probability that the receptor is found bound to ligand *i* in the presence of *n-1* other competing ligands. When there exist different ligands that are experimentally indistinguishable due to the similarity of their binding rates, or when we wish to cluster together ligands of interest and compare their occupancy against the rest of the competing ligands, we replace interactions of any number of ligand types with a single effective type, as follows

7 $$\lambda_{\mathrm{eff}}=\sum_{j}\lambda_{j}$$

8 $$\mu_{\mathrm{eff}}=\frac{\lambda_{\mathrm{eff}}\sum_{j}\mu_{j}P_{j}}{\lambda_{\mathrm{eff}}-\sum_{j}\mu_{j}P_{j}}$$

where λ_eff_ and μ_eff_ are obtained by averaging over any subset of ligands (see Supporting Methods). When determining how to coarse-grain a system that involves many ligands, we can use each ligand’s equilibrium dissociation constant as a guide to ensure that each component is appropriately represented in the average. This approach enables us to categorize ligands into effective types while preserving the competitive interactions among different types.

## Results and Discussion

In the first example of our analysis of competitive PPIs, we utilize a ternary system of competing protein ligands with experimentally determined kinetic and affinity constants. Our test case for the protein receptor–ligand complex is the barnase (Bn) – barstar (Bs) pair, respectively.^38^ Here, Bn is a small 110-residue RNase,^39^ and Bs^38^ is its high-affinity 89-residue ligand.^40^ The primary motivation for this choice is that the Bn-Bs complex has been extensively explored under various experimental conditions and subjected to extensive mutagenesis analysis.^39,41,42^ In addition, PPIs mediated by this receptor–ligand complex are unimodal, exhibiting single populations of dissociation time constants,^25^ thereby facilitating accurate evaluations of the interaction kinetics and dynamics. Specifically, we employed three protein ligands: a strong-affinity Bs, a medium-affinity E76A Bs, and a weak-affinity D39A Bs.

For brevity, these protein ligands are henceforth denoted as L_1_, L_2_, and L_3_ (Table 1). The three protein ligands are identical in structure and composition, except for a key point mutation in the binding site, resulting in significantly different affinities against the same Bn receptor. Their kinetic and affinity constants are listed in Table 1. Using our queuing model applied to three ligands, eq 3, we calculated receptor occupancy, *O*, which is the fraction of time the receptor spends bound to any one of the ligands. Figures 1–2 display three-dimensional (3D) surface plots of receptor occupancy, along with their corresponding topographic contour maps, where the concentration of a strong-affinity (Figure 1) and weak-affinity (Figure 2) competing protein ligand is maintained at a constant value, while the concentrations of the other two ligands are varied. For completeness, Supporting Figure S1 and Table S1 present results obtained by keeping the concentration of the medium-affinity ligand, L_2_, fixed. In Figure 1, the concentration of the strong-affinity ligand L_1_, [L_1_], is maintained at a constant level. At low-nanomolar concentrations of the strong-affinity protein ligand, [L_1_], we obtain a concave 3D surface in which receptor occupancy *O* varies nonmonotonically with respect to changes in the concentrations of competing ligands (Figure 1a). At [L_2_] = [L_3_] = 0 and [L_1_] = 10 nM, we obtain an *O* of 0.13, which corresponds to a noncompetitive PPI with the single protein ligand L_1_. By introducing and gradually increasing the concentration of either one or both of the remaining ligands at fixed [L_1_], we observed a decrease in *O*, followed by an upswing at higher ligand concentrations.

**Table 1:** Kinetic Rate Constants and Affinity Parameters of the Barstar (Bs) Protein Ligands Interacting with Barnase (Bn)^a^

protein ligand	notation	*k*_on_ (10^7^ M^–1^ s^–1^)	*k*_off_ (s^–1^)	*K*_D_ (nM)
Bs	L_1_	1.48	0.95	64
E76A Bs	L_2_	0.32	3.6	1100
D39A Bs	L_3_	0.20	327	168,000

a *k*_on_ and *k*_off_ are the rate constants of association and dissociation, respectively. *K*_D_ denotes the equilibrium dissociation constant. These values were previously determined using single-channel electrical recordings^43^ and an engineered Bn-containing nanopore.^33^

**Figure 1:**
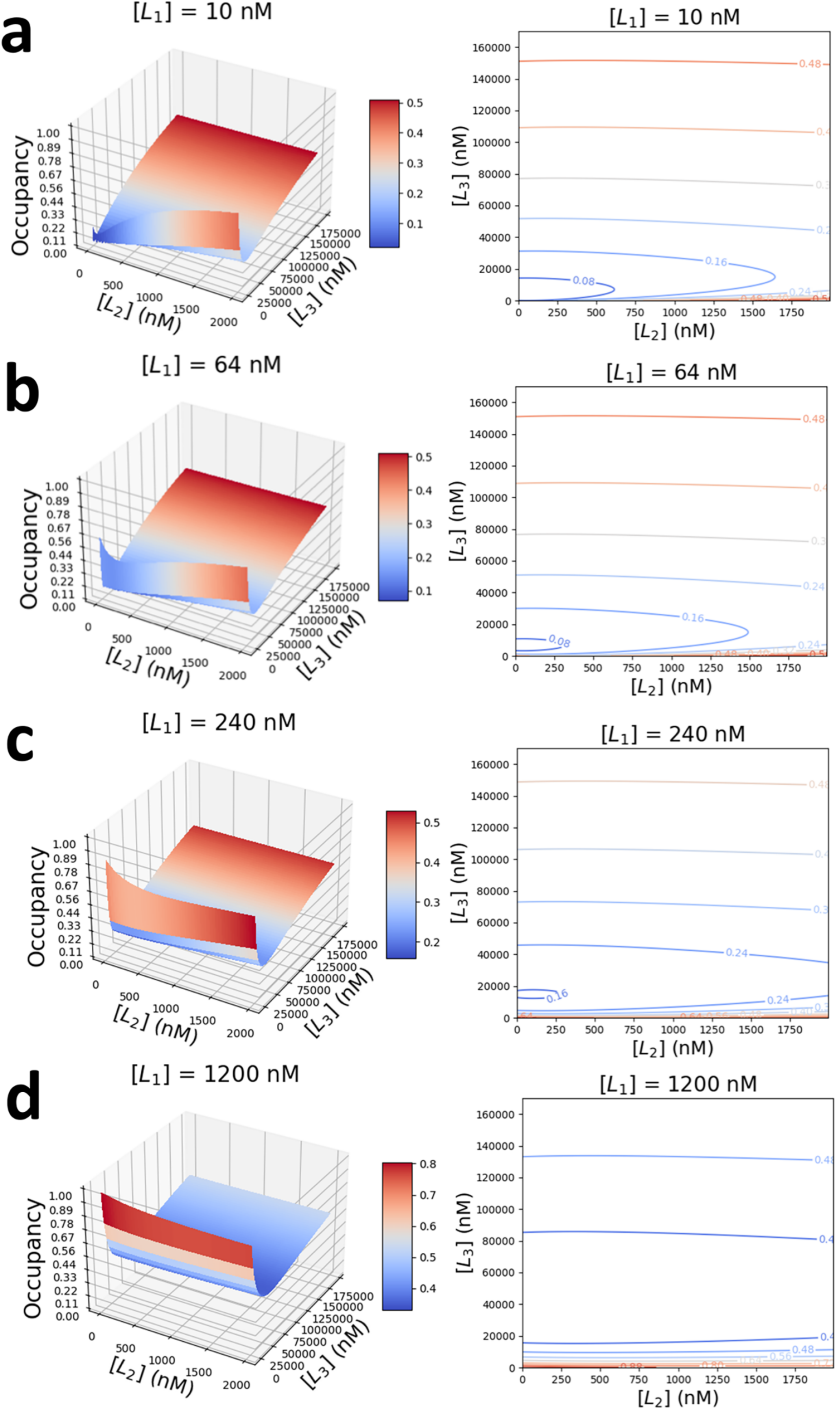
3D surface plots (left) and corresponding contour maps (right) of receptor occupancy, *O*, when exposed to a ternary mixture of competing protein ligands. They are the strong-affinity L_1_, the medium-affinity L_2_, and the weak-affinity L_3_. Plots are obtained for a fixed concentration of L_1_, [L_1_]. (a) [L_1_] = 10 nM; (b) [L_1_] = 64 nM; (c) [L_1_] = 240 nM; and (d) [L_1_] = 1200 nM. Concentrations of the medium- and weak-affinity protein ligands, [L_2_] and [L_3_], are varied in the range 0–2 μM and 0–170 μM. The kinetic rate constants of association and dissociation, *k*_on_ and *k*_off_, respectively, and the equilibrium dissociation constants, *K*_D_, of individual protein ligands against the Bn receptor, are listed in Table 1.

**Figure 2:**
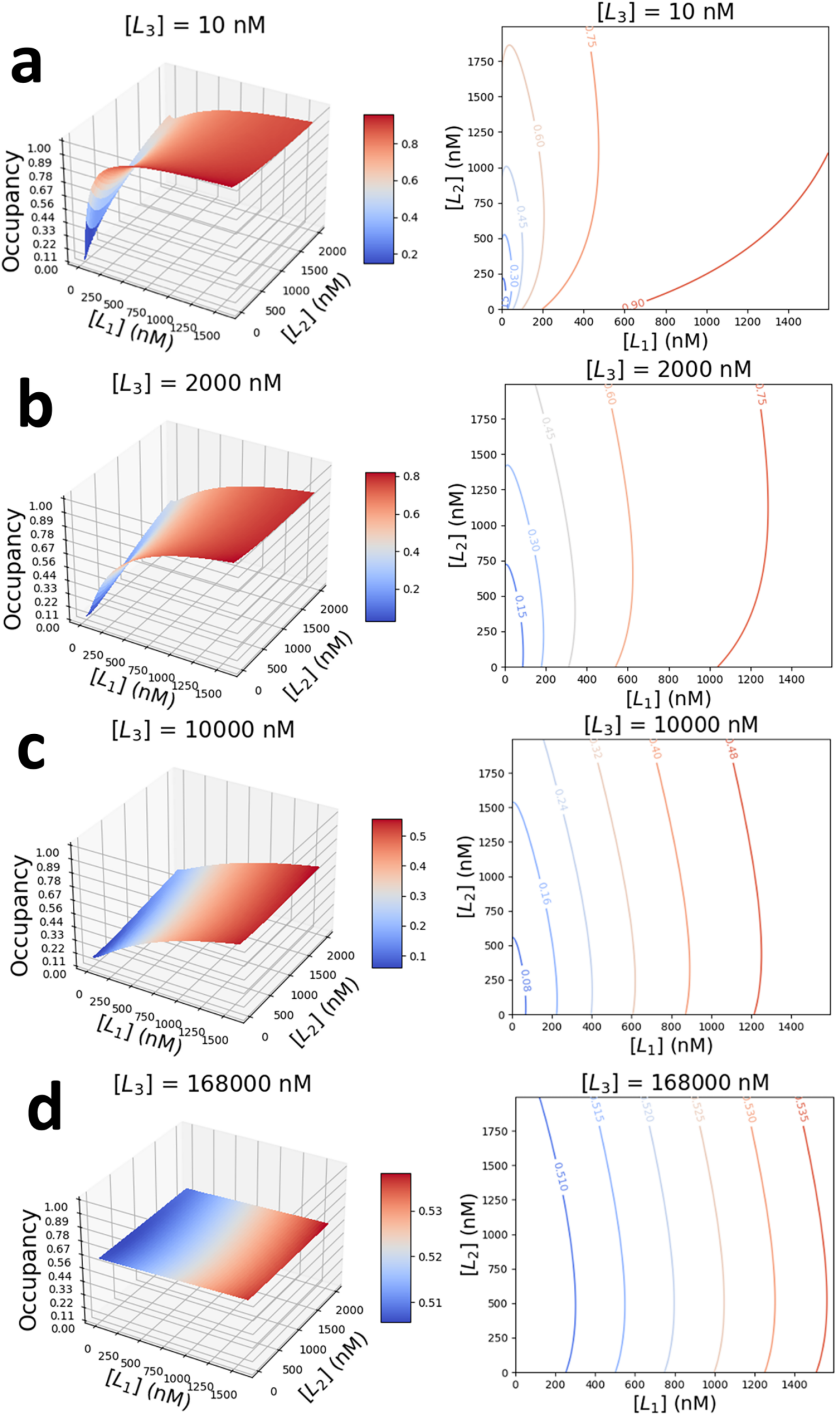
3D surface plots (left) and corresponding contour maps (right) of receptor occupancy, *O*, exposed to a ternary mixture of competing protein ligands. They are the strong-affinity L_1_, the medium-affinity L_2_, and the weak-affinity L_3_. Different plots are obtained for a specific concentration of L_3_, [L_3_]. (a) [L_3_] = 10 nM; (b) [L_3_] = 2 μM; (c) [L_3_] = 10 μM; and (d) [L_3_] = 168 μM. The concentrations of the strong-affinity and medium-affinity protein ligands, [L_1_] and [L_2_], changed in the range 0–1.6 μM and 0–2 μM, respectively. The kinetic rate constants of association and dissociation, and affinity parameters of individual protein ligands against the Bn receptor are displayed in Table 1.

This surprising effect, a drop in receptor occupancy with increasing concentrations of the competing ligand that binds to it, has been observed experimentally at the single-molecule level in binary mixtures of protein ligands^33^ and carries over in ternary mixtures. This result can be explained in terms of competitive PPIs, in which L_1_ has a dominant binding effect on the Bn receptor for very low [L_2_] and [L_3_]. At low ligand concentrations, the system is limited in how often ligands arrive at and subsequently bind to the receptor, which is reflected in the initially low occupancy of 0.13. As ligands with higher dissociation rate constants, *k*_off_, are introduced, they begin competing with L_1_ for the Bn receptor. However, since their *k*_off_ values are much larger, the receptor remains bound to them for a shorter period, effectively resulting in a drop in overall receptor occupancy (see Supporting Methods).^33^ As the concentrations of competing ligands further increase, so do their arrival rates, which leads to an increase in occupancy. Since ligand L_2_ has a much lower dissociation constant than L_3,_ it starts contributing to an increase in occupancy at a concentration that is 2 orders of magnitude lower than that of L_3_ (Supporting Table S2).

By increasing the concentration of L_1_ (Figure 1b–d), we observe two effects: (1) a higher presence of L_1_ increases receptor occupancy, as expected, which shifts the 3D surfaces up toward higher values, and (2) introducing a low-affinity ligand L_3_ at high L_1_ sharply reduces overall receptor occupancy. Even when L_1_ is dominant and saturates the receptor at occupancies close to 1, a small concentration of a medium-affinity ligand, due to competitive binding, can substantially lower this value (Supporting Table S2). In an unusual twist, a receptor that is saturated by binding to the high-affinity ligand can be made again available by introducing small amounts of weak-affinity ligands into the system (Figure 1d).

In contrast, Figure 2 shows occupancy curves when the concentration of the weak-affinity protein ligand L_3_, [L_3_], was kept constant and concentrations of the strong-affinity and medium-affinity ligands, [L_1_] and [L_2_], were varied across ranges 0–1.6 μM and 0–2 μM, respectively. As expected, a low [L_3_] value of 10 nM has a negligible impact on the receptor occupancy. For example, at [L_1_] = [L_2_] = 0, the receptor occupancy due to L_3_ binding was 6.1 × 10^–5^ (Figure 2a). In this example, the addition of higher-affinity ligands increases receptor occupancy. As a result of this, at nonzero concentrations [L_1_] and [L_2_], receptor occupancy is determined by the competitive PPIs between ligands L_1_ and L_2_.

However, when the concentration of the weak-affinity ligand, [L_3_], increases to a micromolar range, receptor occupancy due to L_3_ binding events increases, and higher-affinity ligands compete for the remaining fraction of receptor time. As a result, the local curvature of the 3D surface spanned by [L_1_] and [L_2_] changes in the direction of the increase of both ligands’ concentrations (Figure 2).

This suggests that increases in the concentration of the weakest-affinity ligand can modulate pairwise competitive PPIs between [L_1_] and [L_2_]. Indeed, we observe the biphasic structure of receptor occupancy when [L_2_] is varied for a fixed concentration of [L_1_] = 1000 nM, at two markedly different concentrations of [L_3_]. Specifically, Figure 3 shows a decrease in receptor occupancy with an increase in [L_2_] for fixed values of [L_1_] = 1000 nM and [L_3_] = 10 nM (red curve). This is contrasted with an increase in receptor occupancy as [L_2_] increases for fixed values of [L_1_] = 1000 nM and [L_3_] = 10,000 nM (blue curve). Therefore, significant amplification of weak-affinity protein ligands in complex mixtures can be used to modulate competitive interactions between existing components.

**Figure 3:**
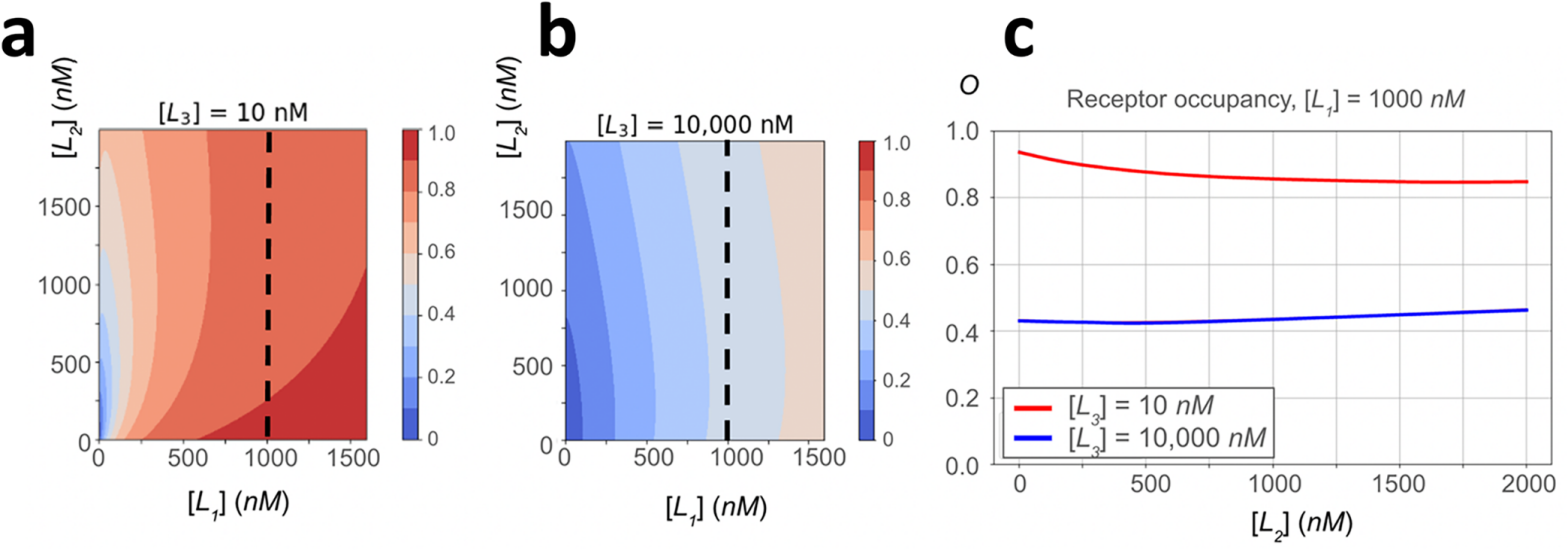
Topographical contour maps (left and center) for two fixed concentrations of weak-affinity L_3_ and the corresponding overall receptor occupancy (right), *O*, as a function of [L_2_] at fixed [L_1_]. (a) The contour map at [L_3_] = 10 nM. (b) The contour map at [L_3_] = 10,000 nM. (c) Receptor occupancies for (a) and (b). A change in surface curvature along a slice at a fixed [L_1_] suggests that the presence of [L_3_] modulates pairwise competitive interactions between the two highest-affinity ligands and the receptor. The receptor occupancy exhibits markedly different behaviors in the two regimes, which are set by the concentration of [L_3_]. The kinetic rate constants of association and dissociation, and affinity parameters of individual protein ligands against the Bn receptor are displayed in Table 1.

In the second example, we examined competitive PPIs of WDR5, a chromatin-associated protein hub with five 14-residue mixed lineage leukemia (MLL/SET1) peptide ligands through the WDR5 interaction (Win) binding site.^4,44^ For simplicity, we name these peptide ligands using the nomenclature of the corresponding full-length proteins, namely MLL2, MLL3, MLL4, SETd1A, and SETd1B. Their kinetic and affinity constants are displayed in Table 2. These interactions mediate the formation of large, multisubunit enzymatic complexes for histone 3 lysine 4 (H3K4) methylation.^45,46^

**Table 2:** Kinetic Rate Constants and Affinity Parameters of the WDR5 Protein Hub with Five Mixed Lineage Leukemia (MLL/SET1) Peptide Ligands^a^

peptide ligand	*k*_on_ (10^5^ M^–1^ s^–1^)	*k*_off_ (10^–3^ s^–1^)	*K*_D_ (nM)
MLL2	3.7	12	33
MLL3	4.9	9	19
MLL4	2.1	41	190
SETd1A	3.1	110	350
SETd1B	3.4	24	69

a *k*_on_ and *k*_off_ are the rate constants of association and dissociation, respectively. *K*_D_ denotes the equilibrium dissociation constant. Values were determined by surface plasmon resonance (SPR), in which WDR5 was immobilized on the surface of the chip sensor.^4,44^

The equilibrium dissociation constants of the MLL/SET1 peptide ligands span an order of magnitude, with MLL2, MLL3, and SETd1B exhibiting medium to strong affinities with WDR5, while MLL4 and SETd1A display comparably medium affinities. Therefore, to analyze this system, we considered a binary mixture of ligands that we generated by coarse-graining the system into two effective ligand types. Ligand A is comprised of MLL2, MLL3, and SETd1B, which were averaged according to the eqs 7–8, while ligand B is obtained by averaging over MLL4 and SETd1A.

Figure 4 shows a topographical receptor occupancy map overlaid with regions where ligand A competes with ligand B for dominance over the receptor. Along the dashed curve, partial occupancies are given with eq 6, of ligand A and ligand B are exactly matched; as we move away from this curve by increasing the concentration of one of the ligands, that ligand’s presence starts competitively reducing the other ligand’s partial occupancy of the receptor. This analysis highlights how partial occupancy, resulting from the binding of a specific ligand to the receptor, can be promoted or suppressed through the arrival of additional isoforms with similar or markedly different binding affinities.

**Figure 4:**
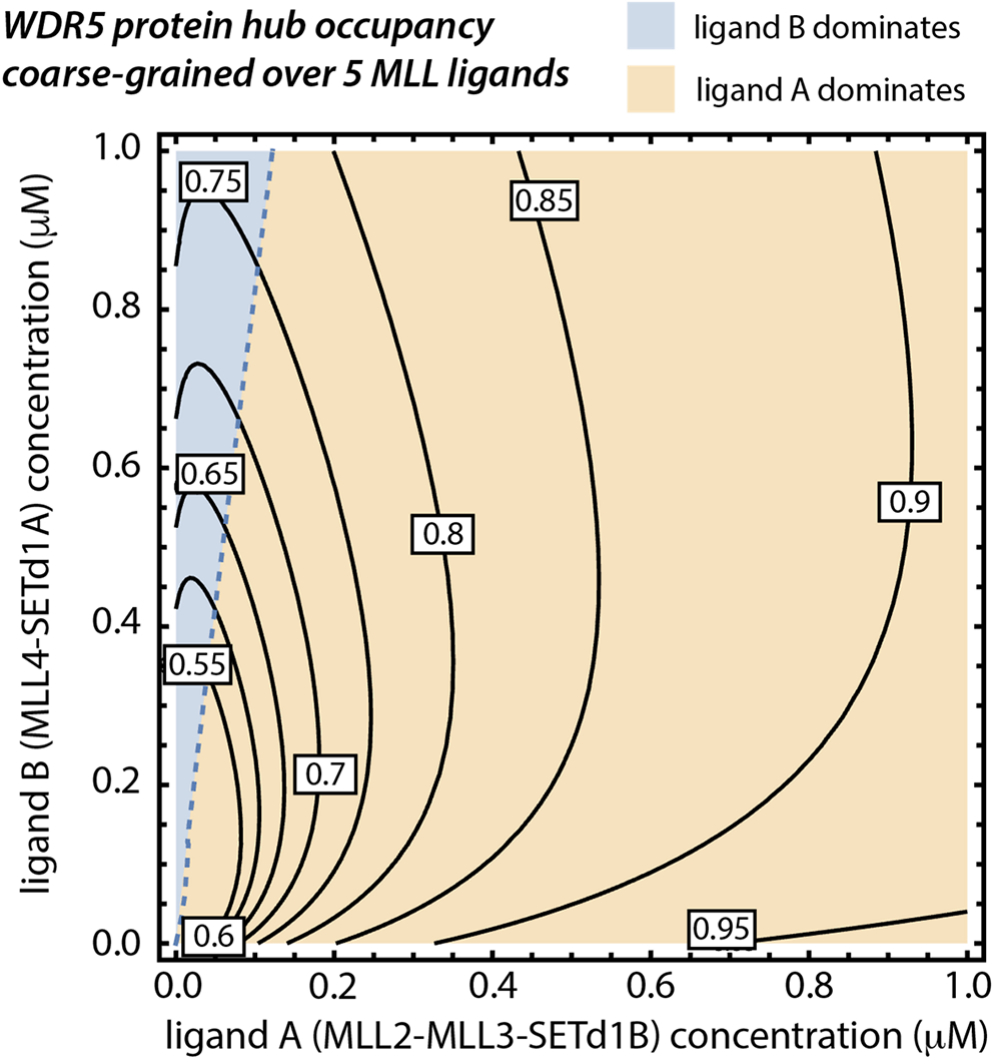
Contour maps of the Win occupancy, *O*, of the WDR5 protein hub exposed to a pentameric mixture of competing protein ligands, grouped into two coarse-grained effective ligands. They are the strong-affinity MLL2, MLL3, and SETd1B, represented by the effective ligand A, and the medium-affinity MLL4 and SETd1A, represented by the effective ligand B. Receptor occupancy values are noted on the corresponding contours. Two regions separated by the dashed curve correspond to areas where one or the other effective ligand type is dominant in binding to the hub. The kinetic rate constants of association and dissociation, and affinity parameters of individual MLL/SET1 peptide ligands against WDR5 are displayed in Table 2.

In the third example, we considered a binary mixture in which competitive PPIs are mediated by a medium-affinity ligand, L_2,_ and a low-affinity ligand, L_3,_ in the Bn-Bs system, whose kinetic and affinity parameters are listed in Table 1. Here, let us take a different approach to the analysis. Let us consider that the weak-affinity protein ligand has key functional implications, so that the partial receptor occupancy owing to its PPI is essential for preserving a given function under physiological conditions. Let us also assume that the medium-affinity ligand L_2_ undergoes chemical modifications due to an external chemical imbalance, resulting in a posttranslationally modified (PTM) L*_2_. Here, we aimed to understand the quantitative implications of PTM, specifically whether this alteration amplifies the binding affinity of L_2_ to the level of a strongly interacting protein ligand. For example, let us attribute the kinetic rate constants of the high-affinity protein ligand (L_1_) in Table 1 to L*_2_.

This way, we have again a ternary mixture of three protein ligands: L*_2_, L_2_, and L_3_, whose concentrations are now coupled through the post-translational modification of one of the ligands. We are primarily interested in evaluating the overall receptor occupancy and the partial occupancy due to the weak-affinity ligand L_3_. On one hand, receptor occupancy is a measure of the system’s overall functionality. On the other hand, the binding of L_3_ to the receptor implies its essential role in maintaining the required function. We consider L_3_ to be the weakest link in a functional chain; therefore, if L_3_ interactions are substantially impaired, the protein recognition system does not operate as intended under physiological conditions.

We will set this up in two stages. In the first stage, L_2_ competes with L_3_, so their partial occupancies are determined by their concentrations and kinetic rate constants. L_2_ has a stronger affinity to bind than L_3_, so it is more often found at the receptor. If we attribute the local concentrations of 100 nM to L_2_ and 1 μM to L_3_, then L_2_ will find the receptor more than 14 times more frequently than L_3_. In the second stage, we implement the PTM-driven L*_2_ proteoform as a biomarker for a disease-like condition that emerged from the unmodified L_2_ ligand. Here, *x* denotes the fractional modification of L_2_ that changes to L*_2_, whereas 1 – *x* is the fraction of L_2_ concentration that remains in the system (Figure 5).

**Figure 5:**
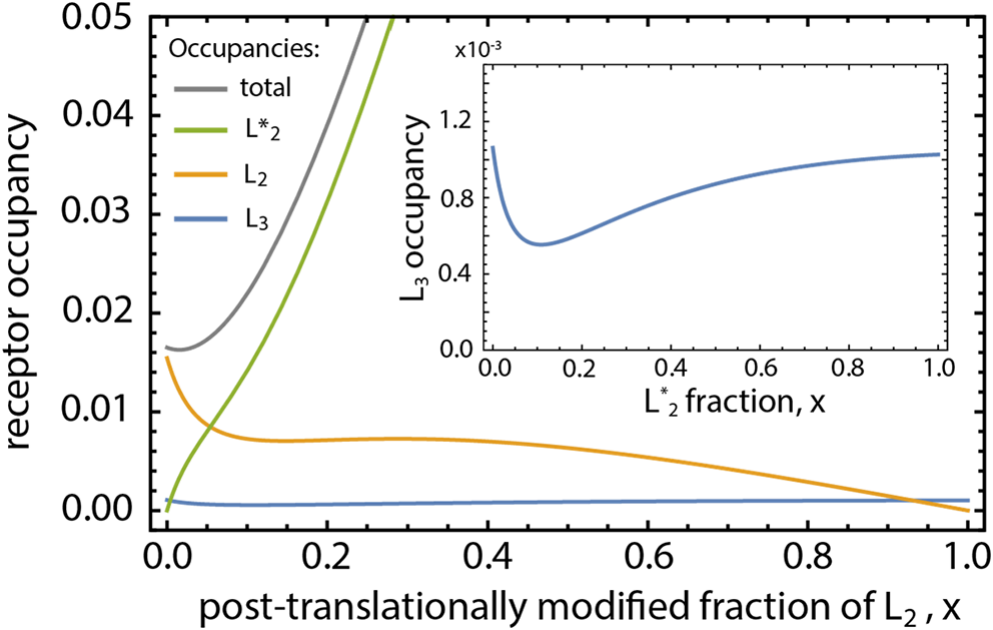
Alterations in the partial and total occupancies of a protein receptor when one protein ligand undergoes a drastic amplification in its binding affinity due to a local post-translational modification. Here, the protein receptor is exposed to a binary mixture of protein ligands L_2_ and L_3_. Variable *x* denotes the fraction of the ligand, L_2_, that is post-translationally modified to L*_2_.

In Figure 5, at intermediate fractional modifications spanning 0 < *x* < 0.1, namely up to 10% of L_2_ is modified, the overall receptor occupancy is slightly changed by the appearance of L*_2_, whose stronger binding affinity does not yet markedly alter the receptor’s availability. However, due to the nonlinear effects in ternary mixtures, we observe a dramatic change in the partial occupancy of L_3_, which, albeit initially small, decreases to 2-fold. While L_3_ in this example is not directly related to L_2_, the apparent decrease in L_3_’s function points to a significant change in the binding dynamics at the receptor, which is, at this stage, difficult to experimentally observe by simply examining overall receptor occupancy. Only later, when *x* grows to values above 0.2, or more than 20% of L_2_ is lost to L*_2_, L_2_ becomes strongly suppressed, while L_3_ starts gaining back its function through an upswing in its partial occupancy. The PTM-driven L*_2_ proteoform hijacks the receptor, resulting in a dramatic increase in overall receptor occupancy from 0.017 to 0.263 as *x* increases from 0 to 1. Yet, it is interesting that at fractional modifications of *x* < 0.1, while there are no adverse or abnormal implications to the receptor availability, L_3_’s reduced binding probability serves as an early messenger of nontrivial dynamical changes in PPI interactions at the receptor. This effect is due to the nonmonotonicity of receptor occupancy in competitive mixtures, a feature of queuing models.

## Conclusions

In summary, we show how a multiligand interacting system has complex implications for the functional features of the receptor. While molecular kinetic models predict reasonably well the frequencies of bound states in well-mixed bulk solutions with plenty of receptors to bind to, here we considered the competitive PPIs in the limit of a single-receptor case. At the single-molecule level, the receptor acts as a bottleneck, as a ligand cannot bind to a receptor that is already occupied. The waiting times resulting from this process generate the competitive PPIs that strongly impact receptor availability, and with it, the molecular binding dynamics. Here, we developed an approach anchored in queuing theory and applied it to highlight different biochemical processes that result from such PPIs. Related queuing approaches have been utilized to model various biological processes, such as metabolic networks,^47^ enzyme kinetics,^48–52^ and multisite gene expression.^53,54^ Our analytical platform is realistically generalizable to numerous protein ligands, anticipating nontrivial aspects and patterns of competitive PPIs within interactomes. This work quantitatively demonstrates the implications of upregulating or downregulating a protein ligand for both partial occupancies by other ligands and overall receptor occupancy. This formalism can also be extended to evaluate PPI inhibitors of a protein hub, which is typically exposed to a complex distribution of numerous ligands. Therefore, it is not surprising that inhibitors targeting hub-directed PPIs may likely affect the overall activity of the receptor and subsets of functional features encoded by those interactions.^5,55^

## Supplementary Material



## Notes

Supporting methods; the Erlang loss model; a binary mixture of protein ligands interacting with a protein receptor; comparison to a steady-state solution; generalization to n-ligands and coarse-graining; supporting tables showing the minimum receptor occupancies; supporting 3D surface plots and corresponding contour maps of receptor occupancy for a ternary mixture of protein ligands (PDF)
