# Supplementary material for: Hub Occupancy by Competitively Interacting Proteins Obeys a Simple Queuing Law
Source: J Phys Chem B. 2025 Sep 24;129(39):9904–12. doi: 10.1021/acs.jpcb.5c04305 (PMC12498412; doi:10.1021/acs.jpcb.5c04305)
Supplement: Supplementary file 1 [file jp5c04305_si_001.pdf]

SUPPLEMENTARY INFORMATION FILE

Hub Occupancy by Competitively Interacting Proteins Obeys A Simple Queuing Law

Yuming Jiang[1], Antun Skanata[1,2]*, and Liviu Movileanu[1,2,3,4]*

[1]Department of Physics, Syracuse University, 201 Physics Building, Syracuse, New York 13244-1130, USA

[2]The BioInspired Institute, Syracuse University, Syracuse, New York 13244, USA

[3]Department of Biomedical and Chemical Engineering, Syracuse University, 329 Link Hall, Syracuse, New York 13244, USA

[4]Department of Biology, Syracuse University, 114 Life Sciences Complex, Syracuse, New York 13244, USA

Running title: Competitive protein-protein interactions utilizing queuing modeling

Keywords: Receptor-ligand interactions; Kinetics; Protein-protein interactions; Interactome; Analytic modeling; Barnase-barstar complex; Mixed lineage leukemia; WD40-repeat protein 5.

*Corresponding authors contact information:
Liviu Movileanu, E-mail: lmovilea@syr.edu
Antun Skanata, E-mail: askanata@syr.edu

Supplementary Methods

1. The Erlang loss model. We utilize a service system to model binding and unbinding events associated with a protein receptor. Ligands randomly arrive at the receptor, transiently bind, and are then released, allowing another ligand to engage with the receptor. Specifically, we formulate a simple model in which the arrival of a ligand to the receptor follows a Poisson process with rate λ. The rate of service, μ, models the rate of release of a captured ligand. Thus, the probability that the receptor is found in a bound state at any moment in time is

$$P = \frac{\lambda}{\lambda+\mu}, \quad \text{(S1)}$$

which is also given in the main text as **Eqn. (1)**. The arrival and service rate in solutions containing a single-ligand type at a concentration c and with k_{on}, k_{off} kinetic rate constants are

$$\lambda = [c]k_{on} \text{ and } \mu = k_{off} \quad \text{(S2)}$$

Derivation of Eqn. (S1). We consider a process of the form $P_0 \leftrightarrow P$, where forward and reverse rates are given with constants λ and μ, and where P_0 is the probability that the receptor is found in an unbound state, and $P = 1 - P_0$ is the probability that the receptor is found in a bound state to a ligand. For this process, we write down the first-order kinetic equation $\frac{dP}{dt} = \lambda P_0 - \mu P = \lambda - (\lambda + \mu)P$. At a late time, its solution approaches $P = \frac{\lambda}{\lambda+\mu}$.

2. A binary mixture of protein ligands interacting with a protein receptor. Here, we distinguish between two ligand types, each of which can carry different rate constants and appear at various concentrations. The protein receptor can thus appear in one of the three possible states: unbound, bound to ligand 1, or bound to ligand 2. The probabilities of observing these states are denoted as P_0, P_1, and

P_2, respectively. The transitions between these receptor states are illustrated in the diagram below, where forward and reverse rates are denoted as λ_i, μ_i for ligand i, with $\lambda_i = [c_i]k_{on,i}$ and $\mu_i = k_{off,i}$.

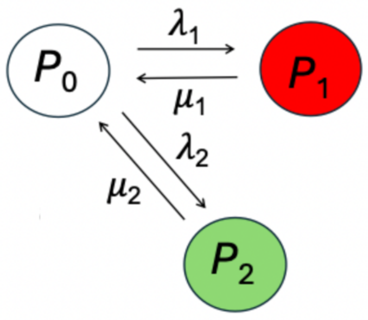

The first-order kinetic equations corresponding to this system are

$$\frac{dP_1}{dt} = \lambda_1 P_0 - \mu_1 P_1$$
$$\frac{dP_2}{dt} = \lambda_2 P_0 - \mu_2 P_2 \qquad \text{(S3)}$$
$$\frac{dP_0}{dt} = \mu_1 P_1 + \mu_2 P_2 - (\lambda_1 + \lambda_2) P_0$$

Solving such and other coupled systems has been the subject of a great deal of work involving combinations of numerical, analytical, and perturbative techniques (e.g., quasi-equilibrium approximations, singular perturbation methods, invariant low-dimensional manifolds). The simplest solution is obtained by assuming steady state, which we comment on further below, and which should work well in bulk when there are plenty of receptors to bind to. However, at the single-molecule level where there is just one receptor that can be either bound or not, we take a different approach, which ultimately reduces to a simple queuing law presented in the main text.

We introduce rescaled variables $y_1 = {}^{P_1}/_{P_0}$ and $y_2 = {}^{P_2}/_{P_0}$ that satisfy

$$\frac{dy_1}{dt} = \lambda_1 - \mu_1 y_1 - y_1 \frac{1}{P_0}\frac{dP_0}{dt}$$
$$\frac{dy_2}{dt} = \lambda_2 - \mu_2 y_2 - y_2 \frac{1}{P_0}\frac{dP_0}{dt} \qquad \text{(S4)}$$

The coupling between y_1 and y_2 is contained in the term proportional to ${}^{dP_0}/_{dt}$. In a quasi-steady state, the fluxes at the receptor are balanced, ${}^{dP_0}/_{dt} = 0$, and the system of equations, **Eqn. (S4),** decouples. Thus, we can solve for y_1 and y_2 independently, as separate processes, which are now reduced to equations for single, non-interacting ligands whose steady-state solutions are given with **Eqn. (S1).** At late times solutions $y_i = {}^{\lambda_i}/_{\mu_i}$ set relative fluxes for each process. The flux balance condition at the receptor ${}^{dP_0}/_{dt} = 0$ is then used to constrain P_0,

$$\mu_1 P_1 + \mu_2 P_2 = (\lambda_1 + \lambda_2) P_0. \qquad \text{(S5)}$$

By inserting P_i through **Eqn. (S1)** of the non-interacting ligands, we obtain an expression for receptor occupancy

$$\mathcal{O} = 1 - P_0 = 1 - \frac{\frac{\mu_1\lambda_1}{\mu_1+\lambda_1} + \frac{\mu_2\lambda_2}{\mu_2+\lambda_2}}{\lambda_1+\lambda_2}. \qquad \text{(S6)}$$

It is easy to check that $0 < \mathcal{O} < 1$, by rewriting

$$\mathcal{O} = \frac{\lambda_1}{\lambda_1+\lambda_2} \cdot \frac{\lambda_1}{\mu_1+\lambda_1} + \frac{\lambda_2}{\lambda_1+\lambda_2} \cdot \frac{\lambda_2}{\mu_2+\lambda_2} = \frac{\lambda_1}{\lambda_1+\lambda_2} \cdot P_1 + \frac{\lambda_2}{\lambda_1+\lambda_2} \cdot P_2 < 1 \qquad \text{(S7)}$$

We distinguish the non-interacting quantities, denoted with P, and the quantities that contain information about competitive PPIs, denoted as $\mathcal{O}$. Note that the probabilities $P_i < 1$ are obtained for single ligand types and therefore do not account for competitive interactions; in this model, competitive interactions arise through the flux balance constraint, **Eqn. (S5)**, which ensures that the total occupancy is less than 1, even if P_1 and P_2 can independently add to a value greater than 1.

This approach to solving the system, **Eqn. (S4),** by inserting the free states to obtain the interacting states, is reminiscent of perturbative expansions around weak potentials. In this system, the coupling is

proportional to $y_i \, {}^{dP_0}\!/_{dt}$. By relaxing the flux balance condition to ${}^{dP_0}\!/_{dt} \ll 1$, the solution can be further improved order by order in perturbation expansion around an appropriate small parameter.

It will be helpful to rewrite **Eqn. (S6)** in terms of partial occupancies $\mathcal{O} = \mathcal{O}_1 + \mathcal{O}_2$, where $\mathcal{O}_1$ is the probability that the receptor is found bound to ligand 1, and likewise for $\mathcal{O}_2$, in a binary mixture of competing ligands. Proportional fluxes along each branch constrain partial occupancies:

$$\frac{\mu_1}{\lambda_1}\mathcal{O}_1 = \frac{\mu_2}{\lambda_2}\mathcal{O}_2 \tag{S8}$$

Using **Eqn. (S8)** where $\mathcal{O} = \mathcal{O}_1 + \mathcal{O}_2$ we find

$$\begin{aligned} \mathcal{O}_1 &= \frac{\lambda_1\mu_2}{\lambda_1\mu_2+\lambda_2\mu_1}\mathcal{O} \\ \mathcal{O}_2 &= \frac{\lambda_2\mu_1}{\lambda_1\mu_2+\lambda_2\mu_1}\mathcal{O} \end{aligned} \tag{S9}$$

Here, partial occupancy of one ligand is influenced by the presence of another that competes for the same receptor. A non-trivial result occurs when we consider a case $\mu_2 \to \infty$, that is, when L_2 does not remain bound to the receptor. In this limit, we find $\mathcal{O}_2 \to 0$ and $\mathcal{O}_1 \to \frac{\lambda_1}{\lambda_1+\lambda_2}P_1$. Here, receptor occupancy is lowered by the nonzero binding affinity of L_2 even though partial occupancy of L_2 vanishes. We find support for this result in single-molecule experiments in binary mixtures of weak and strong affinity ligands, L_1 and L_3. Since the weak affinity ligand has an *off* rate that is orders of magnitude larger and an *on* rate that is an order of magnitude smaller than the strong affinity ligand (**Table 1**), in binary mixtures of L_1 and L_3, almost all of the total receptor occupancy results from binding to L_1.[1]

We compare this to receptor occupancy in a single ligand solution of L_1: from **Eqn. (S1)** and single-ligand measurements, we expect to observe an occupancy slightly over 0.5 at 68 nM L_1. However, the measured occupancy in binary mixtures at 68 nM L_1 and 684 nM L_3 is 0.257, approximately half as much, consistent with a value of 0.22 obtained using **Eqn. (S6).** Therefore, we interpret this result as

indicating that the presence of L_3 lowers total receptor occupancy, without actually occupying the receptor to a significant extent: the partial occupancy of L_3 is orders of magnitude lower than that of L_1.[1] Partial occupancies that we obtained with **Eqn. (S9)** are consistent with other limiting cases, for example, if ligand 2 is removed by taking $\lambda_2 \to 0$, we recover single-ligand occupancy for ligand 1, **Eqn. (S1)**: $\lim_{\lambda_2 \to 0} \mathcal{O}_1 = P_1$.

3. Comparison to a steady-state solution. We consider a solution of **Eqn. (S3)** at the fixed point, with zero flux along each branch $\lambda_i P_0 = \mu_i P_i$. This reduces to a set of equations $\lambda_1 P_0 = \mu_1 P_1,\ \lambda_2 P_0 = \mu_2 P_2,\ P_0 + P_1 + P_2 = 1$, whose solution is $P_0 = \frac{\mu_1\mu_2}{\mu_1\mu_2+\lambda_1\mu_2+\lambda_2\mu_1}, P_1 = \frac{\lambda_1\mu_2}{\mu_1\mu_2+\lambda_1\mu_2+\lambda_2\mu_1}$ and $P_2 = \frac{\lambda_2\mu_1}{\mu_1\mu_2+\lambda_1\mu_2+\lambda_2\mu_1}$. Thus obtained occupancy is a monotonic function of ligand concentration, as $-\frac{dP_0}{d\lambda_i} > 0$ for all values of λ_i. Therefore, this simplest model does not achieve the non-monotonic behavior of receptor occupancy in binary mixtures.[1] Furthermore, by taking the limit $\mu_2 \to \infty$, we obtain $P_0 = \frac{\mu_1}{\mu_1+\lambda_1}, P_1 = \frac{\lambda_1}{\mu_1+\lambda_1}, P_2 = 0$, the binding probabilities in a single-ligand solution. Ligand 2 in this limit no longer perturbs receptor occupancy P_1.

4. Generalization to n-ligands and coarse-graining. Receptor occupancy, **Eq. (S6)** can be readily generalized by considering any number of ligands competing for the receptor, as follows:

$$\mathcal{O} = 1 - \frac{\frac{\mu_1\lambda_1}{\mu_1+\lambda_1}+\frac{\mu_2\lambda_2}{\mu_2+\lambda_2}+\cdots+\frac{\mu_n\lambda_n}{\mu_n+\lambda_n}}{\lambda_1+\lambda_2+\cdots+\lambda_n}, \tag{S10}$$

or in terms of partial occupancies,

$$\mathcal{O} = \mathcal{O}_1 + \mathcal{O}_2 + \cdots + \mathcal{O}_n, \tag{S11}$$

where

$$\frac{\mu_1}{\lambda_1}\mathcal{O}_1 = \frac{\mu_2}{\lambda_2}\mathcal{O}_2 = \cdots = \frac{\mu_i}{\lambda_i}\mathcal{O}_i = \cdots = \frac{\mu_n}{\lambda_n}\mathcal{O}_n \tag{S12}$$

A general solution for the *i*-th partial occupancy is:

$$\mathcal{O}_i = \frac{\lambda_i \prod_{j\neq i}\mu_j}{\sum_{i=1}^{n}\lambda_i \prod_{j\neq i}\mu_j}\mathcal{O} \tag{S13}$$

For example, in the three-ligand case, we obtain:

$$\mathcal{O}_1 = \frac{\lambda_1\mu_2\mu_3}{\lambda_1\mu_2\mu_3 + \lambda_2\mu_1\mu_3 + \lambda_3\mu_1\mu_2}\mathcal{O},$$

$$\mathcal{O}_2 = \frac{\lambda_2\mu_1\mu_3}{\lambda_1\mu_2\mu_3 + \lambda_2\mu_1\mu_3 + \lambda_3\mu_1\mu_2}\mathcal{O}, \tag{S14}$$

$$\mathcal{O}_3 = \frac{\lambda_3\mu_1\mu_2}{\lambda_1\mu_2\mu_3 + \lambda_2\mu_1\mu_3 + \lambda_3\mu_1\mu_2}\mathcal{O}$$

For *n* ligands, receptor occupancy is an *n*-dimensional surface spanned by ligand concentrations. We coarse-grain this system by replacing interactions of any number of ligand types with a single effective type. By requiring that the occupancy of the system with the effective ligand type exactly reproduces the occupancy of the original system, **Eqn. (S10)**,

$$\mathcal{O} \equiv 1 - \frac{\frac{\mu_1\lambda_1}{\mu_1+\lambda_1} + \frac{\mu_{eff}\lambda_{eff}}{\mu_{eff}+\lambda_{eff}}}{\lambda_1+\lambda_{eff}} = 1 - \frac{\frac{\mu_1\lambda_1}{\mu_1+\lambda_1} + \frac{\mu_2\lambda_2}{\mu_2+\lambda_2} + \cdots + \frac{\mu_n\lambda_n}{\mu_n+\lambda_n}}{\lambda_1+\lambda_2+\cdots+\lambda_n}, \tag{S15}$$

we obtain forward and reverse rates of the effective ligand:

$$\lambda_{eff} = \sum_j \lambda_j \tag{S16}$$

$$\mu_{eff} = \frac{\lambda_{eff}\sum_j \mu_j P_j}{\lambda_{eff} - \sum_j \mu_j P_j} \tag{S17}$$

where the effective forward rate is the sum of arrival rates, and the effective reverse rate is the average unbinding rate, weighted by the binding probability of each type. **Eqn. (S10)** is now recast in terms of an effective type, for example:

$$\mathcal{O} = 1 - \frac{\frac{\mu_1\lambda_1}{\mu_1+\lambda_1} + \frac{\mu_{eff}\lambda_{eff}}{\mu_{eff}+\lambda_{eff}}}{\lambda_1+\lambda_{eff}} \tag{S18}$$

where λ_{eff}, μ_{eff} are obtained by averaging over ligands 2 to n. The rate constants of the effective ligand in this coarse-grained system, **Eqns. (S16) - (S17)**, exist in one of three regimes, set by the equilibrium dissociation constant of the effective ligand:

- *Binding-limited*, $\mu_{eff}/\lambda_{eff} \gg 1$, for $\lambda_{eff} \approx \sum_j \mu_j P_j$
- *Release-limited*, $\mu_{eff}/\lambda_{eff} \ll 1$, for $\lambda_{eff} \gg \sum_j \mu_j P_j$
- *Balanced*, $\mu_{eff}/\lambda_{eff} \approx 1$, for $\lambda_{eff} \approx 2 \sum_j \mu_j P_j$

This ability allows us to group ligands and categorize their functionality into classes, each of which is represented by an effective type. Thus, when determining how to coarse-grain a system that involves many ligands, one needs to take note that each component is appropriately represented in the effective ligand, according to its equilibrium dissociation constant.

Table S1. Values of [L_1] and [L_3] that, when introduced separately into a solution containing L_2 at a concentration [L_2], lead to minima in receptor occupancy, O_{min}. These values support plots in **Supplementary Figure S1**. The kinetic rate constants of association and dissociation, and the affinity parameters of individual protein ligands against the Bn receptor are listed in **Table 1**.

[L_1] (nM)	[L_2] (nM)	[L_3] (nM)	O_{min}
0	10	140	0.002
0	200	2,550	0.030
0	1,100	11,000	0.123
0	2,000	17,100	0.180
<0.3	10	0	0.008
5	200	0	0.143
24	1,100	0	0.474
40	2,000	0	0.618

<u>Table S2.</u> Values of [L_2] and [L_3] that, when separately introduced into a solution containing L_1 at a concentration [L_1], lead to minima in receptor occupancy, O_{min}. These values support plots in **Figure 1**. The kinetic rate constants of association and dissociation and the affinity parameters of individual protein ligands against the Bn receptor are listed in **Table 1**.

[L_1] (nM)	[L_2] (nM)	[L_3] (nM)	O_{min}
10	52	0	0.087
64	274	0	0.353
240	730	0	0.632
1,200	1,963	0	0.867
10	0	1,214	0.015
64	0	5,981	0.069
240	0	14,750	0.159
1,200	0	36,626	0.333

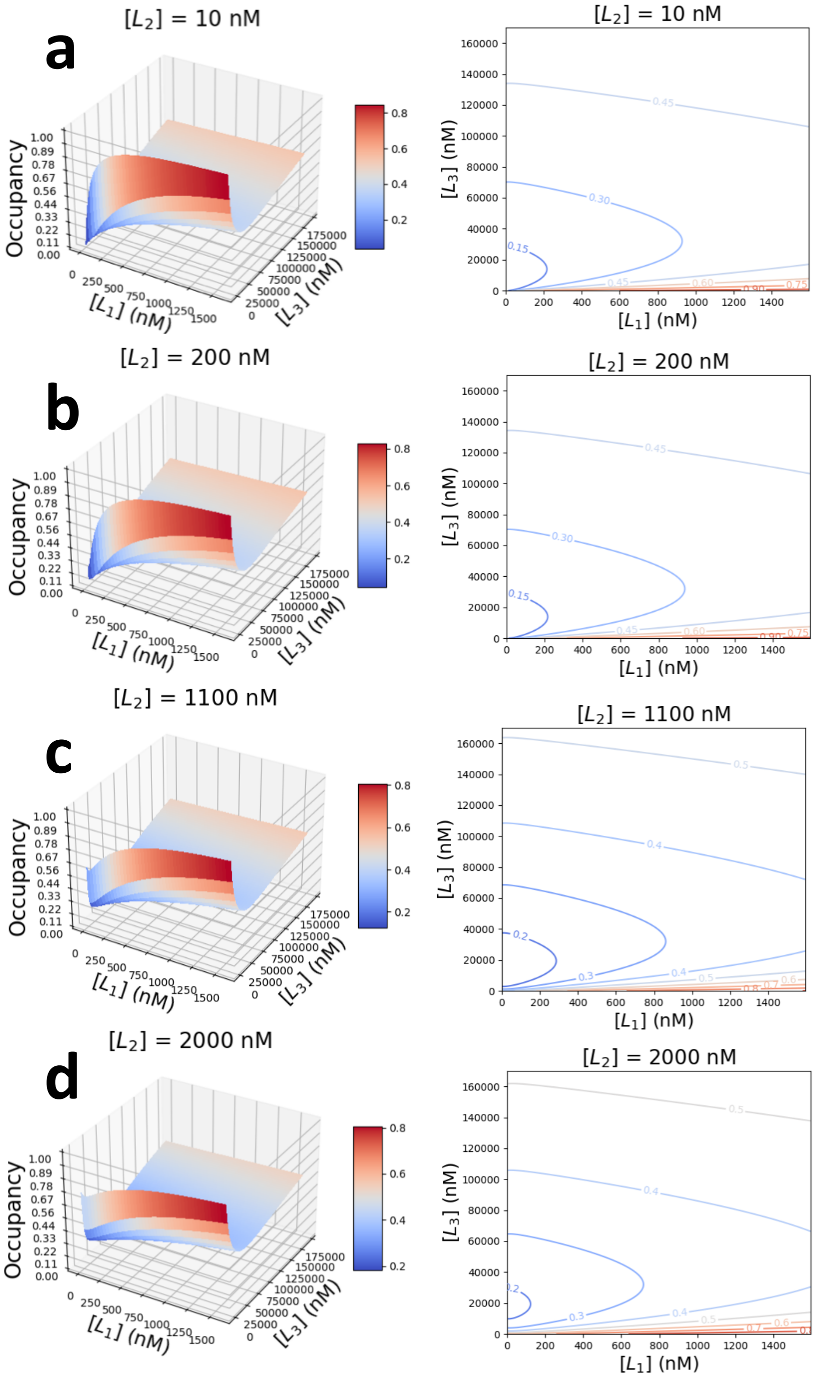

<u>Figure S1.</u> 3D surface plots and corresponding contour maps of the receptor occupancy of Bn when exposed to a mixture of three protein ligands. These are the strong-affinity L_1, the medium-affinity L_2, and the weak-affinity L_3. Plots are obtained for a specific concentration of L_2, $[L_2]$. **(a)** $[L_2]$ = 10 nM; **(b)** $[L_2]$ = 200 nM; **(c)** $[L_2]$ = 1,100 nM; and **(d)** $[L_2]$ = 2,000 nM. The concentrations of the strong-affinity and low-affinity protein ligands, $[L_1]$ and $[L_3]$, changed in the range 0 − 1.6 μM and 0 − 170 μM, respectively. The kinetic rate constants of association and dissociation, and affinity parameters of individual protein ligands against the Bn receptor are displayed in **Table 1**.

REFERENCES

(1) Sun, J.; Skanata, A.; Movileanu, L. Single-Molecule Observation of Competitive Protein-Protein Interactions Utilizing a Nanopore. *ACS Nano* **2025**, *19* (1), 1103-1115. DOI: 10.1021/acsnano.4c13072 From NLM.
